# Goniolactone C, a Styryl Lactone Derivative, Inhibits PDGF-BB-Induced Vascular Smooth Muscle Cell Migration and Proliferation via PDGFR/ERK Signaling

**DOI:** 10.3390/molecules191219501

**Published:** 2014-11-26

**Authors:** Lan Sun, Rui Zhao, Xi Lan, Ruoyun Chen, Si Wang, Guanhua Du

**Affiliations:** 1 Institute of Materia Medica, Chinese Academy of Medical Science and Peking Union Medical College, 1 Xian Nong Tan Street, Beijing 100050, China; 2 Beijing Key Laboratory of Drug Targets Identification and Drug Screening, Beijing 100050, China; 3 State Key Laboratory for Bioactive Substances and Functions of Natural Medicines, Beijing 100050, China; 4 Department of Anesthesiology and Critical Care medicine, School of Medicine, Johns Hopkins University, Baltimore, MD 21205, USA

**Keywords:** styryl lactones, goniolactone C, VSMC, proliferation, migration, PDGF-BB

## Abstract

Platelet-derived growth factor-BB (PDGF-BB) and its downstream effector, extracellular signal-regulated kinase 1/2 (ERK1/2) MAP kinase, initiate a multitude of biological effects, including vascular smooth muscle cell (VSMC) proliferation and migration, which are critical events in the initiation and development of restenosis following percutaneous transluminal coronary angioplasty (PTCA). Styryl lactones are natural products that have been demonstrated to possess anti-proliferative activities. Goniolactone C is a styryl lactone derivative that was first extracted from *Goniothalamus cheliensis* Hu. In the present study, we investigated the effects of goniolactone C on VSMC migration and proliferation. We found that goniolactone C preferentially interacted with cellular systems that rely on PDGF signaling but not those that rely on epidermal growth factor (EGF) and basic fibroblast growth factor (bFGF) signaling. Goniolactone C strongly inhibited PDGF-BB-induced VSMC migration and proliferation. goniolactone C-mediated inhibition of VSMC proliferation was associated with cell cycle arrest, while goniolactone C-mediated inhibition of VSMC migration was associated with the suppression of adhesion molecule expression. In addition, goniolactone C directly inhibited PDGFR-β kinase activity, thereby blocking the downstream effector of PDGF-BB. Thus, the results of the present study suggest a novel adjunctive pharmacological strategy that may be used to prevent angioplasty-related restenosis.

## 1. Introduction

Percutaneous transluminal coronary angioplasty (PTCA) is a standard procedure that is used to restore blood flow in coronary heart diseases, such as angina pectoris and atherosclerosis-induced cardiac infarction. However, the increased proliferation and migration of vascular smooth muscle cells (VSMCs), which are critical events in the initiation and development of restenosis following PTCA, have limited its application [1].

Platelet-derived growth factor-BB (PDGF-BB), one of the most potent mitogens and chemoattractants for vascular smooth muscle cells (VSMCs), plays a central role in provoking restenosis [2]. Upon binding to PDGF-Rβ on VSMCs, PDGF-BB initiates a multitude of biological effects through the activation of extracellular signal-regulated kinase 1/2 (ERK1/2) MAP kinase. ERK1/2 transduces mitogenic signals to the nucleus by phosphorylating and activating specific transcription factors [3]. Therefore, targeting PDGF signaling is a key pharmacological strategy that is used to prevent restenosis and inhibit VSMC proliferation and migration.

**Figure 1:**
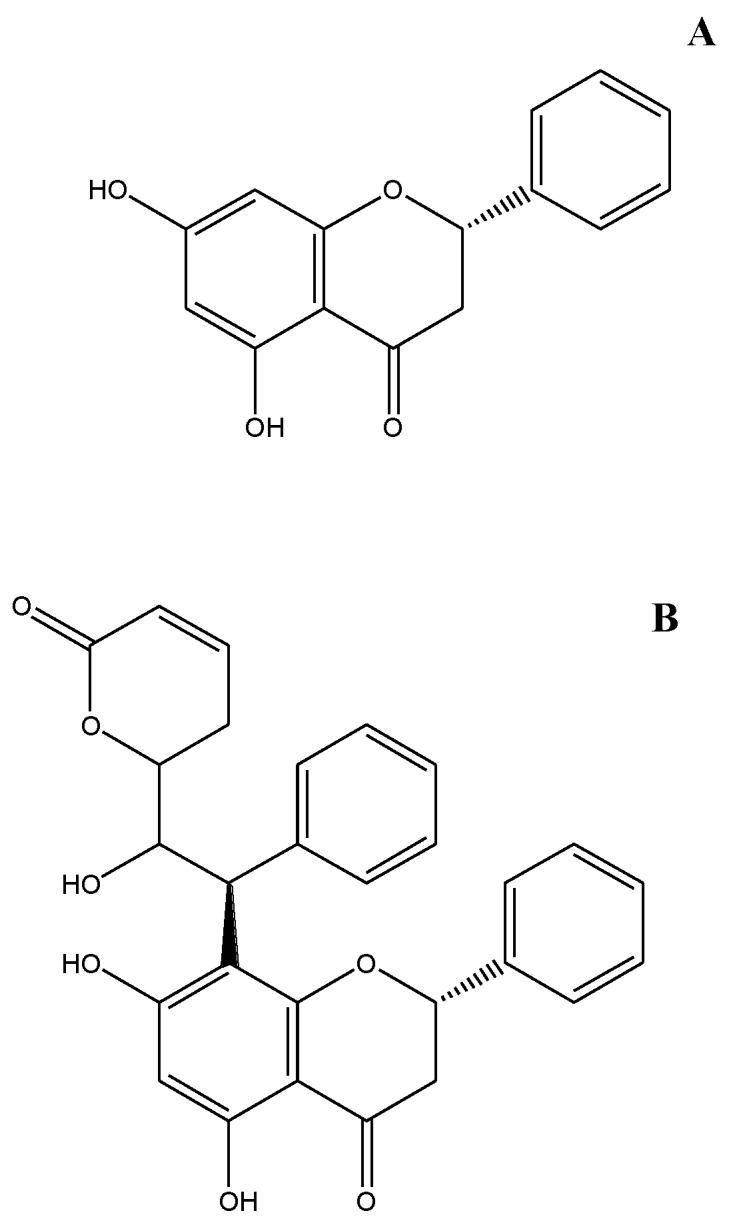
Chemical structures of goniolactone C and pinocembrin. (**A**) Goniolactone C. Molecular formula: C_28_H_24_O_7_; molecular weight: 472.16. (**B**) Pinocembrin. Molecular formula: C_15_H_12_O_4_; molecular weight: 256.25.

There is considerable interest in naturally occurring compounds with anti-proliferative effects on VSMCs. Styryl lactones are natural products that have been demonstrated to possess anti-proliferative effects in tumor cells by blocking cell cycle progression at the G1- to S-phase transition [4]. However, the anti-proliferative effects of styryl lactones in VSMCs remain unknown. We previously extracted a styryl lactone derivative, goniolactone C (Figure 1A), also known as (2*S*)-5,7-dihydroxy-8-[(1*S*,2*R*)-2-hydroxy-2-[(2*R*)-6-oxo-2,3-dihydropyran-2-yl]-1-phenylethyl]-2-phenyl-chroman-4-one, from the roots of *Goniothalamus cheliensis* Hu from Yunnan Province in China [5].

In the present study, we investigated the effects of goniolactone C on growth factor-induced rat VSMC proliferation and growth factor receptor activity. We demonstrate the ERK1/2 inhibition-mediated anti-proliferative effects of goniolactone C on VSMCs. In addition, the influence of goniolactone C on VSMC migration was also examined. 

## 2. Results and Discussion

There are four major findings in the present study. First, goniolactone C was found to strongly inhibit PDGF-BB-induced VSMC migration and proliferation. Second, goniolactone C-mediated inhibition of VSMC proliferation was associated with cell cycle arrest. In addition, goniolactone C-mediated inhibition of VSMC migration was associated with the suppression of adhesion molecule expression. Fourth, goniolactone C directly inhibited PDGFR-β kinase activity and blocked the downstream effector of PDGF-BB. In summary, our results demonstrate that the styryl lactone derivative, goniolactone C, significantly inhibits VSMC proliferation and migration via PDGF-BB/ERK cascade inhibition. Although the model used to study vascular remodeling *in vitro* in the present study was a preclinical model, it may be helpful for the initial evaluation of novel therapeutic approaches.

### 2.1. Goniolactone C Specifically Reduces PDGF-BB-Induced VSMC Proliferation and Migration

**Figure 2:**
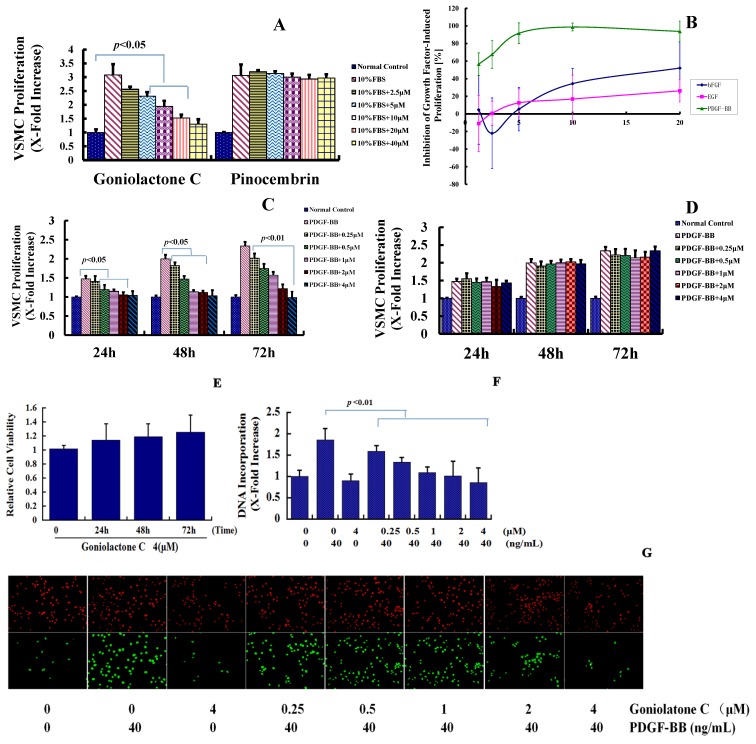
Goniolactone C specifically reduces PDGF-BB-induced VSMC proliferation. (**A**) Goniolactone C but not Pinocembrin inhibits serum-induced VSMC proliferation. Proliferation was expressed as an x-fold increase compared to normal control VSMCs that were incubated in the absence of FBS. (**B**) Goniolactone C attenuates PDGF-induced VSMC proliferation. (**C**,**D**) Goniolactone C (C) but not Pinocembrin (D) inhibits PDGF-BB-induced VSMC proliferation in both a time- and concentration-dependent manner. (**E**) Goniolactone C is not cytotoxic in VSMCs. Confluent VSMCs were treated with 4 μM goniolactone C for 24, 48 and 72 h. (**F**) Goniolactone C inhibits PDGF-BB-induced VSMC DNA synthesis by usding BrdU incoporation assay. (**G**) The green fluorescence (bottom) represents BrdU positive cells, DAPI was employed to detect the nuclei (upper). The data are expressed as the means ± SEM and are representative of three independent experiments.

Goniolactone C, a styryl lactone derivative (Figure 1A) that was extracted from the roots of *Goniothalamus cheliensis* Hu, is structurally related to the flavone pinocembrin (a natural flavone that is found in honey, Figure 1B). In the present study, we compared the inhibitory effects of goniolactone C and pinocembrin on serum-induced VSMC proliferation. As depicted in Figure 2A, goniolactone C inhibited serum-induced VSMC proliferation in a concentration-dependent manner, with a maximum reduction of 86% at a concentration of 40 μM/L. The IC_50_ (the concentration required for 50% growth inhibition) of goniolactone C was 3.94 μM/L under the present experimental conditions. The flavonoid pinocembrin did not affect VSMC proliferation, suggesting that the styryl lactone moiety in goniolactone C represents the pharmacofore. In fact, a preliminary SAR analysis of goniolactone C should be conducted in the future to confirm this assumption PDGF, basic fibroblast growth factor (bFGF), and epidermal growth factor (EGF) are important growth factors that trigger neointima formation following angioplasty. We hypothesized that goniolactone C may act on cellular pathways that are activated by one or more of these growth factors. Thus, we measured the effects of goniolactone C on PDGF-, bFGF-, and EGF-induced VSMC proliferation *in vitro*. As shown in Figure 2B, goniolactone C suppressed PDGF-activated VSMC proliferation in a concentration-dependent manner (IC_50_ 1.82 μM/L), whereas an approximately 10-fold higher concentration of goniolactone C was required to lead to a 50% reduction in EGF/insulin- and bFGF-induced VSMC proliferation, indicating preferential interaction of goniolactone C with cellular systems that rely on PDGF signaling.

We then compared the inhibitory effects of goniolactone C and pinocembrin on PDGF-BB-induced VSMC proliferation. As depicted in Figure 2C,D, goniolactone C inhibited PDGF-BB-induced VSMC proliferation in both a concentration- and time-dependent manner within a concentration range of 0.25 to 4 μM/L for 24–72 h. The flavonoid pinocembrin did not affect VSMC proliferation. When quiescent cells were treated with goniolactone C (4 µM) for 24–72 h in the absence of PDGF-BB, no significant differences were observed in the viability of VSMCs compared to untreated cells, suggesting that goniolactone C is not cytotoxic at the concentrations tested (Figure 2E). 

Styryl lactones are a group of ubiquitous secondary metabolites in the genus *Goniothalamus* that have been demonstrated to possess interesting biological properties, in particular antiproliferative activity and cytotoxicity against cancer cells [4,6,7]. In the present study, cellular activity determinations demonstrated that goniolactone C displays no cytotoxicity in VSMCs. We speculate that the flavonoid group may reduce the cytotoxic activity of goniolactone C. Further investigation is required to determine the mechanism by which this occurs.

We then examined the influence of goniolactone C on PDGF-BB-induced cellular DNA synthesis using a BrdU incorporation assay in VSMCs. As shown in Figure 2F,G, incubation with PDGF-BB for 24 h led to an approximately 2-fold increase in the DNA incorporation rate in VSMCs. Compared to PDGF-BB alone, pretreatment with goniolactone C for two hours blunted PDGF-induced DNA incorporation in a concentration-dependent manner. Hence goniolactone C preferential interacts with cellular systems that rely on PDGF signaling and then inhibits VSMC proliferation.

### 2.2. Goniolactone C-Mediated Inhibition of VSMC Proliferation Is Associated with Cell Cycle Arrest

**Figure 3:**
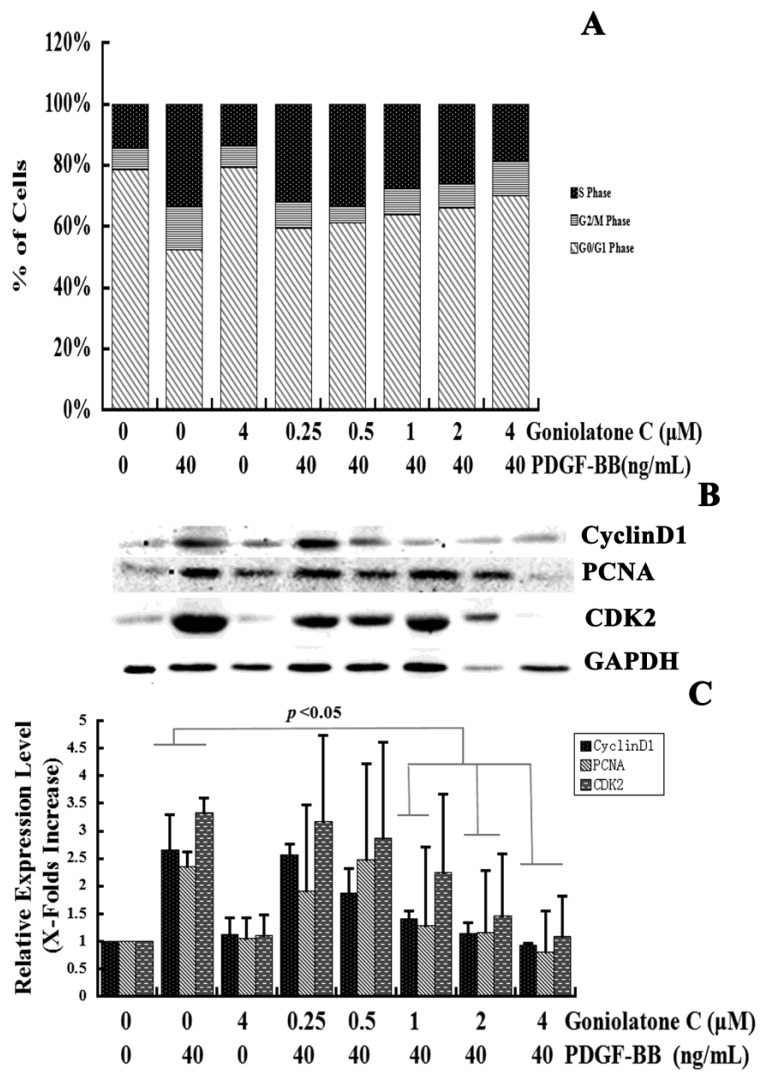
Goniolactone C induced G_0_/G_1_ phase arrest during cell cycle progression. VSMCs were pre-incubated in the presence or absence of goniolactone C in serum-depleted medium for 12 h. The cells were then stimulated with 40 ng/mL of PDGF-BB for 24 h. (**A**) Individual nuclear DNA content was determined by measuring the fluorescence intensity of incorporated propidium iodide. Each value is derived from a representative experiment where data from at least 10,000 events were obtained. The data are expressed as the mean values from three independent experiments. (**B**,**C**) Western blot analyses were performed using antibodies specific for cyclin D1, CDK2 and PCNA. GAPDH was used for normalization. The relative levels of these proteins are expressed as x-fold increases compared to that of the normal control group.

To identify the effects of goniolactone C on cell cycle progression, the proportion of cells in various phases of the cell cycle was analyzed using flow cytometry. VSMCs were pretreated with 0.25–4 µM goniolactone C and were then incubated with PDGF-BB for 24 h. The cells were then harvested and subjected to flow cytometric analyses. As shown in Figure 3A, treatment of PDGF-induced VSMC with goniolactone C caused a concentration-dependent increase in the G_0_/G_1_ phase cell population and a concomitant decrease in the S phase cell population. 

Serum deprivation of VSMCs for 24 h resulted in an approximately 15% synchronization of the cell cycle in S phase. PDGF-BB treatment of the VSMCs for 24 h increased the S phase population to 38%. Pretreatment with goniolactone C at concentrations ranging from 0.25 to 4 μM significantly reduced the S phase cell population to approximately 32% (*p* < 0.05), 33% (*p* = 0.11), 27% (*p* < 0.01), 26% (*p* < 0.01), and 19% (*p* < 0.01), respectively, in PDGF-BB-stimulated cells (Figure 3A). However, PDGF-BB treatment of VSMCs for 24 h reduced the G_0_/G_1_ phase population to 47.33%. Pretreatment with goniolactone C at concentrations of 0.25, 0.5, 1, 2 and 4 μM increased the G_0_/G_1_ phase cell population to approximately 59% (*p* < 0.01), 61% (*p* < 0.01), 63% (*p* < 0.01), 66% (*p* < 0.01), and 70% (*p* < 0.01), indicating that goniolactone C could effectively inhibit DNA synthesis by acting during early cell cycle events. The accumulation of cells in G_0_/G_1_ phase implied a specific effect of goniolactone C on cell cycle progression*.* In addition, the highest concentration of goniolactone C alone did not obviously alter the proportion of cells in various phases of the cell cycle, which indicated that goniolactone C alone does not affect the cell cycle in quiescent VSMCs and that pretreatment with goniolactone C likely impairs the response capacity of PDGFRβ for PDGF-BB stimulation.

Cell cycle progression is strictly controlled by positive and negative regulators that act at checkpoints throughout the cell cycle. CDK2 and CyclinD1 are important positive regulators that boost cell cycle progression from G_0_/G_1_ to S phase, thereby promoting proliferation. In the present study, goniolactone C induced G_0_/G_1_ phase cell cycle arrest. We then detected the expression levels of CDK2 and CyclinD1. As is shown in Figures 3B,C, pretreatment with goniolactone C at concentrations of 1, 2, and 4 μM significantly reduced the expression levels of CyclinD1 (46.7%, 57%, and 65%) and CDK2 (32%, 56% and 67%, *p* < 0.05) as compared to PDGF-BB stimulated VSMCs (*p* < 0.05). Therefore, goniolactone C may regulate the expression of G_0_/G_1_-checkpoint proteins, which is in accordance with the results of our cell cycle analyses. Thus, goniolactone C-mediated inhibition of VSMC proliferation is associated with cell cycle arrest.

### 2.3. Goniolactone C Inhibits PDGF-BB-Induced VSMC Migration and Adhesion Molecule Expression

VSMC migration is another factor underlying neointima formation [8]. The effects of goniolactone C on VSMC migration, which represents another crucial process in the pathogenesis of neointima formation, were also investigated. Both the scratch wound assay and the modified Boyden chamber assay were used. 

In the wound closure/scratch assay, which determines non-directional migratory activity due to the loss of neighboring cells, we observed a VSMC migration-inhibiting activity of goniolactone C within a concentration range from 0.25 to 4 μM. Similarly, in the modified Boyden chamber assay (Transwell migration assay), which involves the migration of cells through a microporous filter toward a high concentration of chemoattractant in a well below, PDGF-BB-induced VSMC migration was also inhibited by goniolactone C in a concentration-dependent manner. 

**Figure 4:**
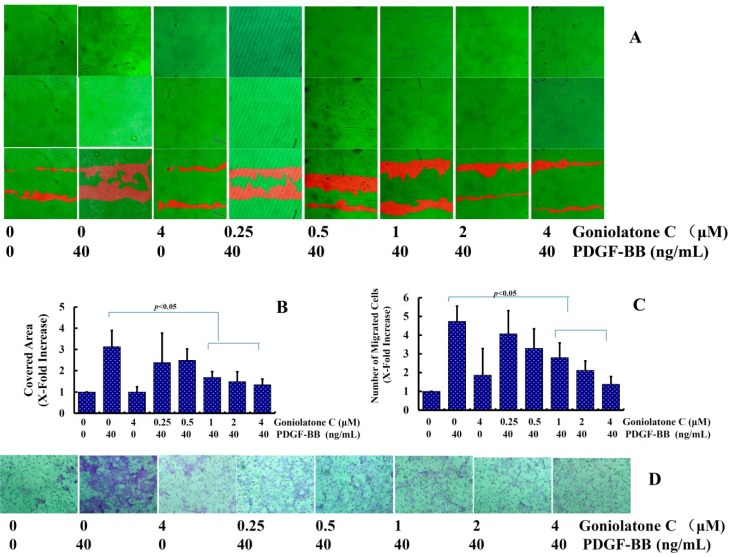

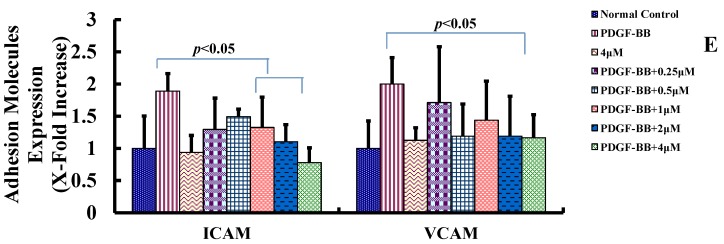
Goniolactone C inhibits PDGF-BB-induced adhesion molecule expression and VSMC migration. VSMCs were treated with goniolactone C at different concentrations (0–4 μM) for 12 h and were then incubated with PDGF-BB for another 12 h. (A and B) A wound-Healing assay was used to determine VSMC migration. Four different fields of migration were photographed using a video camera system and Image Pro-Plus 5.1 Software (Media Cybernetics, Silver Spring, MD, USA) at the intersection of the previously marked line and the wound edge, both prior to and after treatment with PDGF-BB. The migration activity was expressed as the change in the covered area. The samples were run in triplicate in three independent experiments. (**A**) The upper, middle, and bottom images represent the beginning point, end point, and merged images, respectively. The red shadow represents the change in the covered area. (**B**) Change in the covered area. (C and D) A modified Boyden Chamber Assay was applied to measure VSMC migratory ability. (**C**) The number of cells that had migrated to the lower surface per field. (**D**) Cells that had migrated through the 8-μm pores were stained with hematoxylin. (**E**) Levels of VCAM-1 and ICAM-1, as determined using ELISA. The relative levels of these proteins are expressed as x-fold increases compared to the normal control group for three independent experiments.

As is shown in Figure 4A, treatment with PDGF-BB for 12 h led to an approximately 3-fold increase in the basal migration of VSMCs. Compared to PDGF-BB alone, pretreatment with goniolactone C (0.25–4 μM) for 2 h caused a significant reduction in cell migration (Figure 4A,B). In the Transwell assay (Figure 4C,D), treatment with PDGF-BB for 12 h led to an approximately 5-fold increase in the basal migration of VSMCs. Compared to PDGF-BB alone, pretreatment with goniolactone C (0.25–4 μM) for 2 h significantly reduced the number of cells that had migrated. 

The expression levels of several migration regulatory proteins, including intercellular adhesion molecule 1 (ICAM-1) and vascular cell adhesion molecule 1 (VCAM-1), were also detected. As is shown in Figure 4E, PDGF-BB significantly induced the expression of ICAM-1 and VCAM-1. Pretreatment with goniolactone C reduced the expression levels of VCAM-1 and ICAM-1 to nearly normal levels (*p* < 0.05), suggesting that goniolactone C inhibits PDGF-BB-induced VSMC migration by suppressing the expression of migration-related proteins in these cells. The present study demonstrated that goniolactone C significantly inhibited PDGF-BB-induced VSMC migration, which was consistent with the decreases in intercellular adhesion molecule 1 (ICAM-1) and vascular cell adhesion molecule 1 (VCAM-1). These results support the idea that goniolactone C has the potential to inhibit neointima formation.

### 2.4. Goniolactone C Inhibits the PDGFR-β/ERK1/2 Cell Signaling Cascade

PDGF-BB activates PDGFR-β on VSMCs, is one of the most potent mitogens and chemoattractants for vascular smooth muscle cells (VSMCs), and plays a central role in the onset and development of various vascular disorders [9,10,11]. Different PDGFRβ-mediated signaling pathways have been suggested to regulate the proliferative and migratory responses to PDGF-BB, with MAPK being said to regulate proliferation and migration [12].

**Figure 5:**
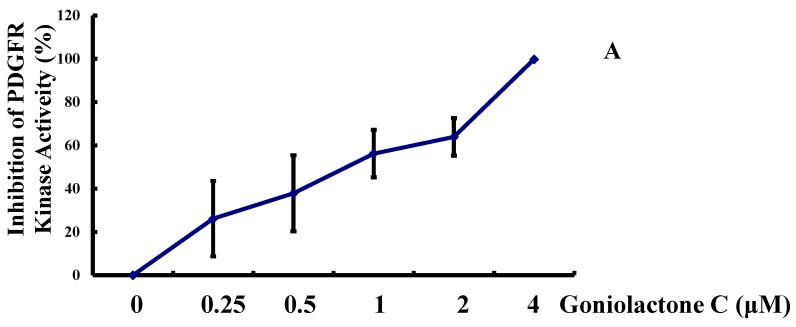

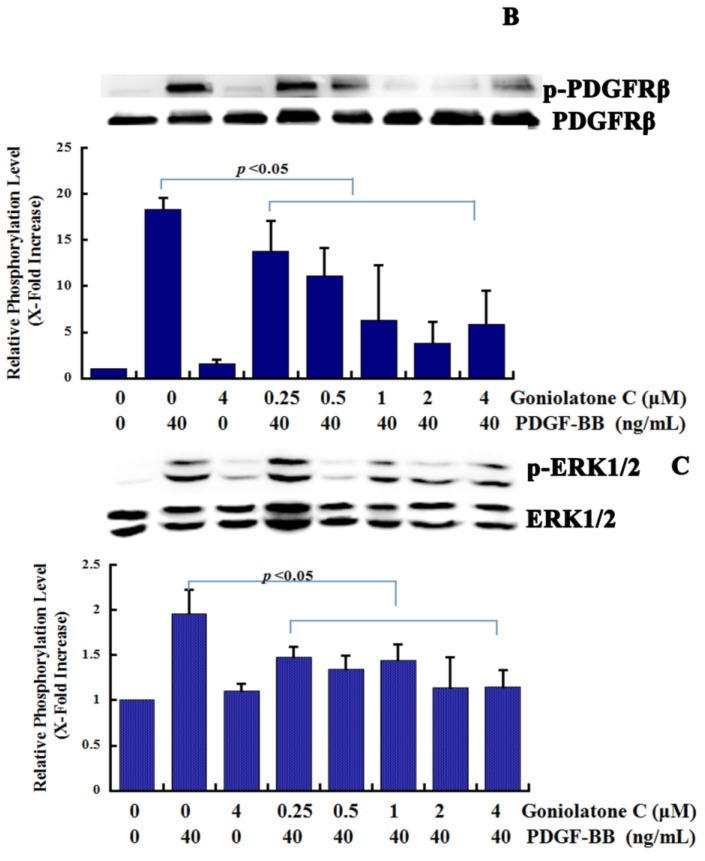
Goniolactone C inhibits the PDGFR-β/ERK1/2 cell signaling cascade. (**A**) A PDGFRβ Kinase Enzyme System assay (PDGFRβ Kinase Enzyme System V3731) was used to measure the *in vitro* activity of PDGFR-β. (**B**,**C**) Confluent VSMCs that had been starved for 24 h in FCS-free DMEM were treated with goniolactone C at different concentrations (0–4 μM) for 2 h and were then incubated in the presence of PDGF-BB for another 30 min. The cells were then lysed, and protein expression was analyzed using 12% SDS-PAGE. Western blot analyses were performed to detect the phosphorylation of PDGFR-β (B) and ERK1/2 (C).

To further delineate the cellular and molecular mechanisms underlying goniolactone C-induced VSMC growth inhibition, we examined the influence of goniolactone C on the PDGF-Rβ activity and ERK1/2 signaling. In the present study, we assumed that the pronounced action of goniolactone C on PDGF-induced VSMC proliferation and migration may be due to the direct interaction of goniolactone C with the PDGF receptor itself. We then analyzed the impact of goniolactone C on the kinase activity of recombinant PDGFR-β by performing a luminescent kinase assay that measures the amount of ADP formed from a kinase reaction; the luminescent signal is positively correlated with the amount of ADP and the kinase activity. We found that goniolactone C diminished human recombinant PDGFR-β autophosphorylation (IC_50_ 0.76 μM/L) in a concentration-dependent manner, as shown in Figure 5A. The high concentration of goniolactone C (4 μM/L) inhibited nearly 100% of PDGFR-β activation. Importantly, goniolactone C also clearly inhibited PDGFR-β activation (autophosphorylation) in VSMCs (Figure 5B), as revealed by measuring PDGFR-β phosphorylation levels. Our results suggest that goniolactone C directly inhibits PDGFR-β activity.

Then we evaluated the effects of goniolactone C on the activation of ERK1/2 signaling cascades because ERK1/2 is the downstream effector kinase of PDGFR and a crucial mediator of VSMC proliferation and migration. After pretreatment with goniolactone C, VSMCs were stimulated with PDGF-BB for 30 min and the phosphorylation status of ERK1/2 MAPK was measured using western blotting analysis. Goniolactone C markedly inhibited PDGF-BB-mediated ERK1/2 activation in a concentration-dependent manner in VSMCs (Figure 5B,C). 

Our results suggested that goniolactone C pretreatment inhibited PDGFRβ kinase activity in a concentration-dependent manner and this inhibitory effect was associated with the inhibition of receptor phosphorylation. Moreover, receptor-triggered downstream ERK1/2 signaling events were blocked. Although the mechanisms underlying the inhibitory effects of styryl lactones remain to be established, previous studies have suggested that some of these molecules may inhibit the activation of receptor tyrosine kinases, resulting in attenuated activation of key signaling intermediates [13]. We hypothesize that a similar mechanism may also be involved in the PDGFRβ inhibitory effects of goniolactone C. However, goniolactone C failed to attenuate PDGF-stimulated JNK and p38 MAPK phosphorylation. Our results suggest that blockage of the PDGFRβ-ERK1/2 pathway is involved in goniolactone C-induced proliferation and migration suppression in PDGF-BB-stimulated VSMCs.

## 3. Experimental Section

### 3.1. General Information

The experimental protocol conformed to the Guide for the Care and Use of Laboratory Animals, which was published by the US National Institutes of Health (NIH Publication No. 85-23, revised in 1996), and was approved by the Animal Studies Committee of Peking Union Medical College (permit number: 002701). The culture of VSMCs, which were derived from the carotid arteries of male Sprague-Dawley rats, was carried out as previously described [14].

### 3.2. Antibodies and Major Reagents

A phospho-PDGFRβ (Tyr 751) mouse mAb (3173), a PDGFRβ rabbit mAb (3169), a phospho-p44/42 MAPK (Erk1/2) (Thr202/Tyr204) (197G2) rabbit mAb (4377), and a p44/42 MAPK (Erk1/2) (137F5) rabbit mAb (4695) were purchased from Cell Signaling Technology (Danvers, MA, USA). A monoclonal mouse anti-BrdU antibody (MS-1058) was purchased from Thermo Scientific (Thermo Scientific, Rockford, IL, USA). A monoclonal mouse anti-glyceraldehyde-3-phosphate dehydrogenase (GAPDH) antibody was purchased from Santa Cruz Biotechnology (Dallas, TX, USA). An isotype-matched fluorescein isothiocyanate (FITC)-conjugated anti-rat IgG1 secondary antibody was purchased from Invitrogen (Camarillo, CA, USA). Goniolactone C was dissolved in DMSO and gradient diluted with DMEM growth medium to reach the appropriate concentrations, stored at 4 °C and used as soon as possible. The final concentration of DMSO in the diluent is less than 1/1000. 4',6-Diamidino-2-phenylindole 2HCl (DAPI) was purchased from BIOMOL (New York, NY, USA). 5-Bromo-2'-deoxyuridine (BrdU, B9285) and 1,1,3,3-tetramethoxypropane (108383) were purchased from Sigma-Aldrich (Sigma-Aldrich, St. Louis, MO, USA). Propidium iodide (PI) was purchased from Invitrogen.

### 3.3. Proliration Assay

#### 3.3.1. Serum-Induced VSMC Proliferation Assay

Serum-induced VSMC proliferation assays were performed. Confluent VSMCs that had been starved for 24 h in FCS-free DMEM were treated with goniolactone C or pinocembrin at different concentrations (0–40 μM) for 12 h and were then incubated in the presence of FBS for 24 h. VSMCs were used and the assay was performed as previously described [14].

#### 3.3.2. Growth Factor-Induced VSMC Proliferation Assay

Confluent VSMCs were treated with goniolactone C at different concentrations (0–40 μM) for 12 h and were then incubated in the presence of PDGF-BB (40 ng/mL), EGF (10 μg/mL), or bFGF (100 ng/mL) for another 24 h. When we detect the time and concentration-dependent effect of goniolactone C on PDGF-BB-induced VSMC proliferation, Confluent VSMCs were treated with goniolactone C at different concentrations (0–4 μM) for 12 h and were then incubated in the presence of PDGF-BB (40 ng/mL) for 24, 48 and 72 h. Crystal staining assay was usded to determin VSMC proliferation rate.

#### 3.3.3. BrdU Incorporation Assay

VSMCs were seeded at approximately 30% confluence in confocal dishes. After 24 h, the cells were starved in FCS-free medium for the next 24 h. The cells were then pre-treated with goniolactone C for 12 h, followed by treatment with PDGF-BB (40 ng/mL) for another 24 h. Five hours prior to harvesting the cells, BrdU was added to the medium (10 μM). After incubation with a mouse anti-BrdU antibody and an isotype-matched fluorescein isothiocyanate (FITC)-conjugated anti-rat IgG1 secondary antibody, propidium iodide (PI) was employed to detect the nuclei. The labeled cells were examined under a Zeiss confocal microscope, and images were obtained using a LEICA TCS SP2 laser scanning confocal microscope (Leica, Wetzlar, Germany).

### 3.4. Kinase Activity Assays

A PDGFRβ Kinase Enzyme System assay (PDGFRβ Kinase Enzyme System V3731) was used to measure the *in vitro* activity of PDGFR-β according to the manufacturer’s recommended instructions. Briefly, after the initial incubation (25 °C, 60 min), 5 μL of ADP‐Glo™ Reagent was added and then incubated at room temperature for 40 min. Kinase Detection Reagent (10 μL) was then added and incubated at room temperature for 30 min. Finally, the luminescence was recorded using a MicroBeta microplate scintillation counter (PerkinElmer, Waltham, MA, USA).

### 3.5. Migration Assays

#### 3.5.1. Wound-Healing Assay

Cell migration was also assessed using wound-healing assays as previously described [9]. Confluent VSMCs that has been starved for 24 h in FCS-free DMEM were used. Four different fields of migration were photographed using a video camera system with Image Pro Plus 5.1 Software (Media Cybernetics, Silver Spring, MD, USA) at the intersection of the previously marked line and the wound edge, both prior to and after treatment with PDGF-BB for 12 h. Migration was expressed as the change in the covered area.

#### 3.5.2. Modified Boyden Chamber Assay

Cell migration was assessed using Corning Transwell chambers (Modified Boyden Chamber) according to the manufacturer’s guidelines. At the endpoint, cells that had migrated through the 8-μm pores were stained with hematoxylin. The number of cells that had migrated to the lower surface of each filter was counted in different fields at a magnification of 200× by three independent observers using Image Pro-Plus 5.0 software (Media Cybernetics, Rockville, MD, USA). The samples were run in triplicate in three independent experiments.

### 3.6. Western Blot Analyses

Immunoprecipitation and immunoblotting were performed. The bands on the films were quantified using Quantity One software (Bio-Rad, Richmond, CA, USA) and normalized to GAPDH, which was used as a loading control.

### 3.7. Cell Cycle Progression Analyses

VSMCs were seeded into 100-mm culture dishes at 1 × 10^5^ cells/mL and grown until they had reached 70% confluence. The medium was then replaced with serum-free media containing goniolactone C. After incubation for 12 h, PDGF-BB (40 ng/mL) was added. Subsequently, the cells were incubated for 24 h, trypsinized, and then centrifuged at 1500*×* *g* for 5 min. The obtained pellets were suspended in 1 mL of 1*×* PBS, washed twice, and re-centrifuged. The pellets were suspended in 70% ethanol and fixed overnight at 4 °C. The cell cycle phase was determined as previously described [4]. The proportions of cells in G_0_/G_1_, S and G_2_/M phases were determined using the computer program odFitLT (Verity Software House, Topsham, ME, USA).

### 3.8. Assessment of ICAM-1 and VCAM-1 Production

VSMCs were pretreated with goniolactone C for 12 h, after which point, PDGF-BB (40 ng/mL) was added and the cells were incubated for 12 h. The supernatants were then collected to determine the levels of ICAM-1 and VCAM-1. Crystal violet staining was used to detect the number of cells. The levels of each cytokine were evaluated using enzyme-linked immunosorbent assay (ELISA) kits (R&D Systems, Minneapolis, MN, USA) according to the manufacturer’s recommendations, and the concentrations were adjusted according to the number of cells.

### 3.9. Statistics

The results are expressed as the means ± SEM. For the *in vitro* experiments, the data were evaluated using one-way analysis of variance (ANOVA) and the Newman-Keuls post-hoc test. *p* ≤ 0.05 was considered to be statistically significant.

## 4. Conclusions

In summary, this study provides evidence that a new styryl lactone derivative, goniolactone C, strongly attenuates VSMC proliferation and migration. Reductions in smooth muscle cell migration and proliferation due to the direct inhibition of PDGFR/ERK signaling cascades is likely involved in these effects. The findings presented here are limited to multi-passaged cells. Future studies are required to confirm the activity of this cascade under *in vivo* conditions. 
